# Spatial suicide clusters in Australia between 2010 and 2012: a comparison of cluster and non-cluster among young people and adults

**DOI:** 10.1186/s12888-016-1127-8

**Published:** 2016-11-22

**Authors:** Jo Robinson, Lay San Too, Jane Pirkis, Matthew J. Spittal

**Affiliations:** 1 Orygen, The National Centre of Excellence in Youth Mental Health, Locked Bag 10, Parkville, VIC 3052 Australia; 2 Melbourne School of Population and Global Health, Level 5, 207 Bouverie Street, Carlton, VIC 3053 Australia

**Keywords:** Spatial suicide cluster, Australia, Youth, Indigenous, Scan statistic

## Abstract

**Background:**

A suicide cluster has been defined as a group of suicides that occur closer together in time and space than would normally be expected. We aimed to examine the extent to which suicide clusters exist among young people and adults in Australia and to determine whether differences exist between cluster and non-cluster suicides.

**Methods:**

Suicide data were obtained from the National Coronial Information System for the period 2010 and 2012. Data on date of death, postcode, age at the time of death, sex, suicide method, ICD-10 code for cause of death, marital status, employment status, and aboriginality were retrieved. We examined the presence of spatial clusters separately for youth suicides and adult suicides using the Scan statistic. Pearson’s chi-square was used to compare the characteristics of cluster suicides with non-cluster suicides.

**Results:**

We identified 12 spatial clusters between 2010 and 2012. Five occurred among young people (*n* = 53, representing 5.6% [53/940] of youth suicides) and seven occurred among adults (*n* = 137, representing 2.3% [137/5939] of adult suicides). Clusters ranged in size from three to 21 for youth and from three to 31 for adults. When compared to adults, suicides by young people were significantly more likely to occur as part of a cluster (difference = 3.3%, 95% confidence interval [CI] = 1.8 to 4.8, *p* < 0.0001). Suicides by people with an Indigenous background were also significantly more likely to occur in a cluster than suicide by non-Indigenous people and this was the case among both young people and adults.

**Conclusions:**

Suicide clusters have a significant negative impact on the communities in which they occur. As a result it is important to find effective ways of managing and containing suicide clusters. To date there is limited evidence for the effectiveness of those strategies typically employed, in particular in Indigenous settings, and developing this evidence base needs to be a future priority. Future research that examines in more depth the socio-demographic and clinical factors associated with suicide clusters is also warranted in order that appropriate interventions can be developed.

## Background

A suicide cluster is typically defined as ‘*a group of suicides or suicide attempts, or both, that occur closer together in time and space than would normally be expected on the basis of either statistical prediction or community expectation’* [1, 2]. This definition is useful although determining the presence or absence of a cluster can be difficult in practice as it is often unclear what constitutes the minimum ‘normal’ number of deaths, over a given time period and particular location [3].

Suicide clusters have typically been investigated using one of two approaches. The first involves identifying a group of suicides that have occurred in a particular area within a relatively short period of time, and mapping the associations between the individuals who have died (e.g. [4, 5]). The second approach relies on quantitative methods that identify statistically greater than expected numbers of suicides occurring in particular locations over specific time periods. Here the time and space dimensions can be varied, creating opportunities for clusters to be identified along both dimensions simultaneously, or by focusing on just one dimension (leaving the other dimension fixed at a constant value).

Despite common perceptions, suicide cluster deaths are relatively rare [6], however when they occur they can have significant negative consequences on the community, largely due to the risk of further suicides, complicated grief reactions and the potential for ongoing trauma [2, 6, 7]. For these reasons it is important to have a clear understanding of the frequency at which clusters occur and the risk factors for suicide clusters among different sectors of the population, for whom effective preventative strategies may be targeted.

Qi and colleagues examined spatial suicide clusters in Australia during the period 1999 and 2003 [8]. They found 13 clusters, which included 12 clusters in males and one cluster in females. These represented 21.6% of all male suicides and 3.0% of all female suicides. Cheung and colleagues examined spatial-temporal suicide clusters occurring in Australia during the period 2004–2008 and identified 15 suicide clusters, which together accounted for 2.4% of all suicides [9].

There is some evidence to suggest that suicide clusters are more common in certain sectors of the population, and in certain settings [10]. For example, international studies report that suicide clusters more frequently occur among young people than adults, with an estimated one to five per cent of teenage suicides in America thought to be part of a cluster [11–13]. Psychiatric inpatient units and prisons have also been identified as common settings for suicide clusters; it has been estimated that around 10% of suicides by people with mental illness and 6% of prison suicides are the result of clustering effects [14, 15]. Suicide clusters in Indigenous communities have been reported in several countries, including Australia [16–18], Canada [19] and the U.S. [20, 21].

However many of the previous studies are descriptive in nature and little is known about the individual level risk factors for being part of a suicide cluster. Cheung and colleagues [18] examined area level predictors of suicide clusters and identified that Indigenous origin, living in remote areas, and living in Queensland, Western Australia and the Northern Territory (three Australian states) were indicators of greater risk.

Whilst previous studies have examined suicide clusters in the general population, to the best of our knowledge no previous study has examined suicide clusters among Australian young people specifically nor compared the characteristics of cluster suicides to non-cluster suicides among Australian young people and adults. Thus the aims of this study are to examine the extent to which suicide clusters exist among both young people and adults in Australia and to determine whether or not differences exist between cluster and non-cluster suicides in both young people and adults.

## Methods

### Suicide data

We obtained data on deaths classified as intentional self-harm from the National Coronial Information System (NCIS [22]). The NCIS, established in 2001, is a national internet-based data storage and retrieval system of Australian coronial records on all reportable deaths. It provides basic information including age, sex, marital status and employment status, and aboriginality collected from coronial files as well as coding of ICD-10 cause of death assigned by the Australian Bureau of Statistics (ABS) [23, 24].

We included deaths recorded as being due to intentional self-harm (ICD-10 codes X60–X84) occurring in Australia between 2010 and 2012 from the database (*n* = 7202). We excluded suicides that occurred outside Australia (*n* = 8); where the deceased’s home was located outside Australia (*n* = 18); with missing information on usual residential postcode (*n* = 33); with unknown month of death (*n* = 221); or with a residential postcode that did not correspond to the 2006 population and coordinate data (*n* = 43). This left a total of 6879 suicides that were included in the analysis. We categorized cases into two groups. They were cases where the deceased was aged 24 years old or less at the time of their death (categorised as young people) and cases where the deceased was aged 25 years old or more (categorised as adult). This cut-off was selected as it has been commonly used to categorize young people and adults [25, 26].

We retrieved the following information for each included case: date of death, postcode of usual residence, age at the time of death, sex, suicide method, ICD-10 code for cause of death, marital status, employment status, and aboriginality. Remoteness for each postcode was classified based on the 2006 Australian Bureau of Statistics (ABS) Geographical Classification Remoteness Structure [27].

### Population and geographical data

We obtained population estimates for all postcodes from the 2006 ABS census data. 2006 data were used because the deceased’s geographical information recorded in the NCIS is based on the 2006 ABS postal areas. The geographical coordinates of the centroids for all postcodes were calculated with ArcGIS software using the Australian digital map file from the ABS for 2006.

### Statistical analysis

We examined the presence of spatial clusters separately for youth suicides and adult suicides. We used the Scan statistics from SaTScan v9.4.1 to detect these clusters. SaTScan is software developed specifically for the spatial, temporal and spatial-temporal scan statistics [28]. As a first step, we calculated the maximum suicide incidence rate within postcodes, which is required to set the value for spatial window. As a result, a rate of 0.026 was obtained for the youth spatial window and a rate of 0.111 was obtained for the adult spatial window. We then used these values for the spatial cluster detection. A circular shape was chosen as the shape of the spatial scan window because this is typically used to detect suicide clusters [8, 9]. This circular window was gradually moved on each centroid point of geographical locations, with each circle reflecting a possible cluster. Our analysis was based at the state/territory level. This meant a separate scan analysis was conducted for each state/territory, and that clusters could not subtend state/territory boundaries.

We selected Poisson discrete model for our approach, as this model is adjusted for the uneven geographical population density. For each possible cluster, their likelihood was assessed using Monte Carlo stimulation [29] and considered to be a “possible cluster” if its p-value was less than 0.10. We classified suicides detected within the areas of possible clusters as cluster suicides and suicides located outside the areas of significant clusters as non-cluster suicides. Pearson’s chi-square test of independence was used to compare the characteristics of cluster suicides with non-cluster suicides. Fisher’s exact probability test was employed when over 20% of cells had expected counts smaller than five.

### Ethics

This study was approved by the Department of Justice Human Research Ethics Committee (CF/14/22880).

## Results

There were 940 (13.7%) youth suicides and 5939 (86.3%) adult suicides included in this study. The national youth suicide rate was 7.2 per 100,000 persons per year and the adult suicide rate was 15.0 per 100,000 persons per year (Table 1). Of the states and territories, the Northern Territory had the highest rate of both youth and adult suicides (30.3 and 22.5 per 100,000 persons per year respectively), followed by Western Australia (11.2 and 20.2) and Queensland (10.0 and 19.0).

**Table 1:** Suicide rates by states and territories in young people and adults

	Young people			Adult		
State/territory	Total cases	Total population	Cases per 100 000 per year	Total cases	Total population	Cases per 100 000 per year
NSW	192	1 403 250	4.6	1635	4 370 145	12.5
QLD	262	872 789	10.0	1457	2 548 959	19.0
VIC	183	1 066 539	5.7	1279	3 304 467	12.9
WA	148	441 131	11.2	775	1 280 958	20.2
SA	75	318 848	7.8	467	1 029 676	15.1
NT	44	48 393	30.3	78	115 412	22.5
TAS	25	101 991	8.2	163	319 870	17.0
ACT	11	76 275	4.8	85	209 918	13.5
National	940	4 329 216	7.2	5939	13 179 405	15.0

### Cluster detection

Our analysis identified 12 spatial clusters (*n* = 190) over the three-year period investigated. This included five clusters among young people (*n* = 53, representing 5.6% [53/940] of all youth suicides) and seven clusters among adults (*n* = 137, representing 2.3% [137/5939] of all adult suicides) (Table 2). The clusters ranged in size from three to 21 cases for youth suicides and from three to 31 cases for adult suicides. When compared to adults, suicides by young people were significantly more likely to occur as part of a cluster (difference = 3.3%, 95% confidence interval [CI] = 1.8 to 4.8, *p* < 0.0001). A range of suicide methods were used in both the adult and the youth clusters.

**Table 2:** Information on spatial clusters of youth suicides and adult suicides

No	States/territories	Number of cases	Expected cases	% of cases^a^	Remoteness	*p*-value
Youth suicides						
1	NSW	11	2	5.7	Major cities/inner regional	0.003
2	QLD	21	5	8.0	Very remote	<0.001
3	VIC	3	0	1.6	Major cities	0.066
4	WA	15	1	10.1	Very remote	<0.001
5	NT	3	0	6.8	Very remote	0.005
Adult suicides						
6	NSW	25	9	3.4	Inner regional	0.014
7	NSW	31	14	3.4	Major cities	0.053
8	VIC	26	8	3.5	Major cities	0.001
9	VIC	19	6	3.5	Major cities	0.011
10	WA	22	7	4.3	Remote/very remote	0.003
11	WA	11	3	4.3	Major cities	0.080
12	NT	3	0	3.8	Outer regional	0.024

^a^Proportion of all suicides accounted for by cluster suicides in each state/territory

Of the five youth suicide clusters, one was located in New South Wales (*n* = 11, 5.7% of youth suicides in the state), one in Queensland (*n* = 21, 8.0%), one in Victoria (*n* = 3, 1.6%), one in Western Australia (*n* = 15, 10.1%), and one in the Northern Territory (*n* = 3, 6.8%). The clusters found in New South Wales and Victoria both occurred in urban areas while the other clusters occurred in remote areas.

**Fig. 1:**
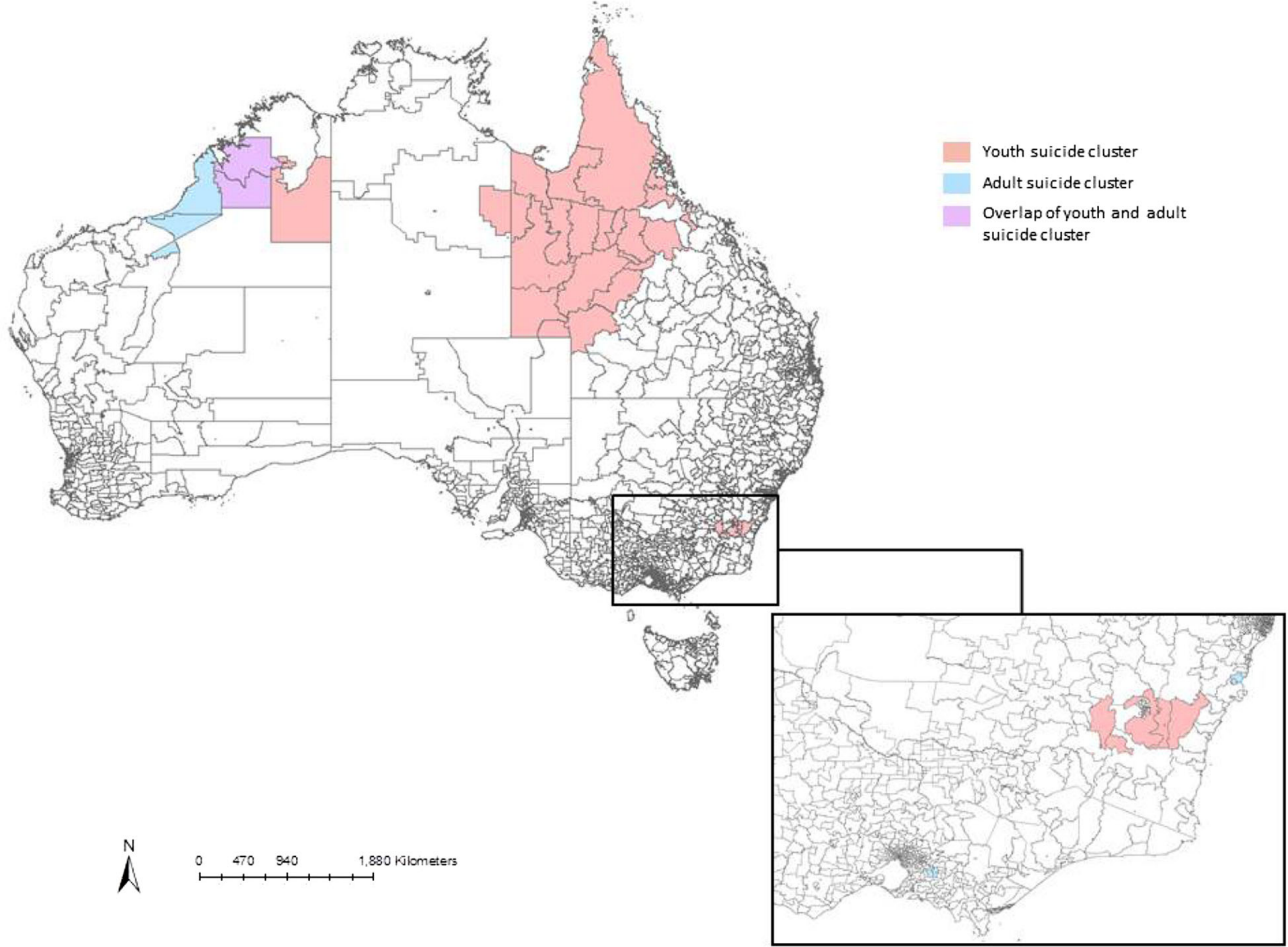
Geographical location of suicide clusters

Of the seven adult suicide clusters, two occurred in New South Wales (*n* = 56, 3.4%), Victoria (*n* = 45, 3.5%) and Western Australia (*n* = 33, 4.3%). The remaining cluster occurred in the Northern Territory (*n* = 3, 3.8%). Please see Fig. 1.

When the proportion of youth and adult suicides that occurred as part of a cluster were compared we found that a higher proportion of youth suicides was accounted for by cluster suicides than was the case for adult suicides in all states and territories, with the exception of Victoria. Victoria was the only state that had a greater proportion of adult cluster suicides relative to youth cluster suicides, although this difference was non-significant (3.5% vs. 1.6%, difference = 1.9%, 95% CI = −0.2 to 4.0, *p* = 0.176).

### Comparison of characteristics of cluster and non-cluster suicides

Among youth suicides, we found that suicides by people with an Indigenous background were significantly more likely to occur in a cluster than suicide by non-Indigenous people (58.5% vs. 13.1%, *p* < 0.001). No other differences between cluster and non-cluster suicides were evident, including in terms of sex, suicide method, marital status and employment status (Table 3).

**Table 3:** Characteristics of cluster and non-cluster cases

	Youth suicide *n* (%)			Adult suicide *n* (%)		
	Cluster case	Non-cluster case	*p*-value	Cluster case	Non-cluster case	*p*-value
Number	53	887		137	5802	
Sex			0.235			0.130
Female	12 (22.6)	269 (30.3)		39 (28.5)	1332 (23.0)	
Male	41 (77.4)	618 (69.7)		98 (71.5)	4470 (77.0)	
Suicide method			0.478^a^			<0.001
Poisoning	1 (1.9)	33 (3.7)		19 (13.9)	865 (14.9)	
Motor vehicle exhaust	2 (3.8)	39 (4.4)		5 (3.7)	505 (8.7)	
Hanging	46 (86.8)	629 (70.9)		69 (50.4)	2956 (51.0)	
Drowning	0	6 (0.7)		3 (2.2)	110 (1.9)	
Firearms	1 (1.9)	19 (2.1)		4 (2.9)	422 (7.3)	
Cutting/piercing	0	7 (0.8)		4 (2.9)	170 (2.9)	
Jumping	0	51 (5.8)		17 (12.4)	244 (4.2)	
Other	2 (3.8)	71 (8.0)		4 (2.9)	305 (5.3)	
Unknown	1 (1.9)	32 (3.6)		12 (8.8)	255 (3.9)	
Marital status			0.246^a^			0.374
Never married	39 (73.6)	669 (75.4)		23 (16.8)	1087 (18.7)	
Widowed/divorced/separated	0	24 (2.7)		48 (35.0)	1764 (30.4)	
Married (including de facto)	10 (18.9)	102 (11.5)		42 (30.7)	2258 (38.9)	
Unknown	4 (7.5)	92 (10.4)		24 (17.5)	693 (11.9)	
Employment status			0.094			0.012
Employed	18 (34.0)	292 (32.9)		50 (36.5)	2344 (40.4)	
Unemployed	20 (37.7)	216 (24.4)		45 (32.9)	1248 (21.5)	
Not in the labour force	11 (20.8)	280 (31.6)		32 (23.4)	1558 (26.9)	
Others (e.g., prisoner)	1 (1.9)	5 (10.6)		2 (1.5)	26 (0.5)	
Unknown	3 (5.7)	94 (10.6)		8 (5.8)	626 (10.8)	
Aboriginality			<0.001			<0.001^a^
Aboriginal/Torres Strait Islander descent	31 (58.5)	116 (13.1)		15 (11.0)	195 (3.4)	
Non-aboriginal/Torres Strait Islander descent	21 (39.6)	676 (76.2)		105 (76.6)	4911 (84.6)	
Unknown	1 (1.9)	95 (10.7)		17 (12.4)	696 (12.0)	

^a^Fisher’s exact test

Among adult suicides, cluster and non-cluster suicides differed in their method of suicide (*p* < 0.001). In particular, suicides identified as being in a cluster were more likely to involve drowning (2.2% vs. 1.9%) and jumping (12.4% vs. 4.2%). Suicides by people who were unemployed were also more likely to be in a cluster (32.9% vs. 21.5%, *p* = 0.012). Finally, as was the case with youth suicides, suicides by Indigenous adults were more likely to be identified as being part of a cluster (11.0% vs. 3.4%, Fisher’s exact test < 0.001). Adult cluster and non-cluster suicides did not differ in terms of either sex or marital status.

## Discussion

### Key findings

This study used the SaTScan statistic in order to examine the spatial of suicide clusters among both young people and adults in Australia during the three-year period 2010 and 2012. It also examined the socio-demographic characteristics of cluster and non-cluster suicides among these two populations.

Overall we identified 12 spatial suicide clusters, which accounted for 190 suicide deaths. This included five clusters among young people (*n* = 53), which represented 5.6% of all youth suicides, and seven clusters among adults (*n* = 137), representing 2.3% of all adult suicides. When compared to adults, suicides by young people were significantly more likely to occur as part of a cluster. This supports previous U.S.-based studies that have found stronger clustering effects among young people than among adults [12, 13] and suggests that interventions designed to reduce the risk of clusters occurring are required, in particular among young people.

Three of the five youth suicide clusters occurred in remote areas, but this was not the case for adult clusters, most of which occurred in major cities.

Few differences were identified between cluster suicides and non-cluster suicides in both populations with the exception of Indigenous status. In both cases, suicides by Indigenous people were significantly more likely to be part of a suicide cluster than suicides by non-Indigenous people. This supports previous literature that has identified Indigenous status as a risk factor for being part of a suicide cluster [18], and earlier studies that described Indigenous communities as a common setting for the occurrence of suicide clusters [17, 19, 20]. This suggests that these settings need to be the focus of future preventative activity.

### Limitations

Before considering the implications of these findings, it is important to acknowledge that this study had some limitations. Firstly, some suicides were removed from the analysis due to missing information regarding month of death and area of residence. As a result, some cluster-related suicides may have been excluded from the analysis.

Secondly, we only had access to a relatively limited number of demographic and suicide-related characteristics, such as age, gender, Indigenous status, area of residence and suicide method. We did not have access to clinical or treatment-related variables such as history of mental illness, hospitalization or previous self-harm. For this reason, we could not compare cluster and non-cluster suicides on these variables. Similarly it was beyond the scope of this study to examine variables such as the presence or absence of mental and physical comorbidity and the presence or absence of life stressors before suicide. This could all be an area for future research.

Thirdly, the methodology employed only allowed the detection of clusters occurring closely together in space, time, and space-time and did not allow the identification of clusters through other mechanisms (e.g., via online social networks). Again, this would be worthy of future investigation.

Finally, our spatial boundaries were constrained by using a circular scan window and within-state analysis. The use of a circular scan meant that we could not detect clusters that were non-circular or of irregular shape such as elliptical shape, which is longer and narrower compared to the circular shape. The use of elliptical shape may reduce the chance of missing clusters that cover linear settlements or areas. By performing within-state analysis, we could not detect clusters that stretched across more than one state or territory.

### Broader implications

In this study we found evidence of five (spatial) suicide clusters among young people and seven among adults making suicides by young people significantly more likely to occur as part of a cluster than adult suicides. We also found that being of Indigenous origin places an individual at higher risk of being part of a suicide cluster.

The development and maintenance of suicide clusters are often explained by theories relating to contagion and imitation, whereby one person’s suicide is thought to influence others to act in the same way [4, 30–32] and those most susceptible to this process are believed to be young people [21]. Others who may also be susceptible are thought to be those who may witness the death itself, and those who may already be vulnerable in some way, such as have a history of mental illness or suicide-related behaviour [33], or have previously been bereaved by suicide [4]. Given the substantially higher rates of psychological distress, hospitalisation for mental illness and exposure to suicide among Indigenous Australians [34–36] it may be hypothesised that they may also be more susceptible to this process.

In terms of interventions designed to help prevent, manage and respond to suicide clusters, national resources have been developed in Australia [37], as well as in other countries including the United States [38] and England [39]. These are not specific to either young people or Indigenous Australians but broadly speaking, they provide a framework designed to help communities to develop a coordinated response or action plan that can be adapted according to local need. For example these may differ according to the community or setting in which a death has occurred and according to who is likely to be involved in the postvention response (e.g. clinicians, family members, religious leaders, prison or school staff). In general these resources recommend the following steps: 1) Ensuring that a community is prepared for a suicide cluster (e.g., by having a community response plan in place together with a team who would be responsible for its implementation); 2) identifying that a cluster is developing via routine monitoring of suicide deaths; 3) responding to the cluster (e.g., by identifying people who require additional support and providing the support or assistance required); and 4) stepping down the response, which should include planning for future significant dates and ongoing monitoring plus longer-term follow-up and evaluation.

Other community responses to managing suicide clusters, often termed ‘postvention responses’, aim to promote recovery after a suicide and prevent further suicide deaths [40]. These activities can be delivered at community level or in specific settings, such as schools. A previous review identified five studies that examined postvention responses to youth suicide clusters specifically [41]; of these two were community-based [42, 43] and three were conducted in school settings [44–46]. None were delivered in Indigenous communities specifically. Six key postvention responses were identified; these included community-level approaches such as the development and implementation of a community response plan, promoting responsible reporting of the deaths by the media, and engaging in community recovery activities; and school-based approaches such as debriefing for students, the provision of counseling, and screening high-risk individuals for signs of trauma and elevated risk.

A final approach used to assist schools manage the aftermath of a student suicide has been the development of postvention guidelines and toolkits. Examples of these are available from Australia and the United States [47–49]. These toolkits generally contain guidance on how to inform students, parents and the wider community of a suicide, how to support students and staff in both the short and longer term, and how to best manage funerals and memorials.

However despite the range of resources, responses and guidelines that have been developed, both in Australia and overseas, to the best of our knowledge evaluation of these types of resource has been limited. As a result there remains limited evidence regarding the effectiveness of interventions designed to either prevent or contain suicide clusters. However rigorous evaluation of these types of strategies, in particular in communities with large Indigenous populations and using appropriate methodologies, is a necessary next step if we are to develop the evidence-based approaches to managing suicide clusters in Australia and elsewhere.

## Conclusions

Suicides among young people more commonly occur as part of a cluster than those by adults. The same can be said of suicides among Indigenous Australians. Suicide clusters have a significant negative impact on the communities in which they occur. Suicide is the leading cause of death among young people and Indigenous young people are between four and five times likely to die by suicide than their non-Indigenous counterparts [36]. As a result it is important to find effective ways of managing suicide clusters in order to minimise the risk of subsequent deaths. To date there is limited evidence for the effectiveness of those strategies typically employed to manage and contain suicide clusters, in particular in Indigenous settings, and developing this evidence base needs to be a future priority. Future research that examines in more depth the socio-demographic and clinical factors associated with suicide clusters is also warranted in order that appropriate interventions can be developed.

The Australian government is currently in the process of reforming the suicide prevention strategy with substantial emphasis being placed upon a refocusing of effort to prevent suicide among Indigenous Australians and on testing regionally-based approaches to suicide prevention [50]. The findings from this study suggest that strategies to manage suicide clusters need to form part of this approach if we are to make an impact on the rates of suicide among young Australians.

## Abbreviations

ABS: Australian Bureau of Statistics
CI: Confidence interval
ICD-10: International Statistical Classification of Diseases and Related Health Problems 10th Revision
NCIS: National Coronial Information System
